# Supplementary material for: Freshwater macrophytes harbor viruses representing all five major phyla of the RNA viral kingdom Orthornavirae
Source: PeerJ. 2022 Aug 16;10:e13875. doi: 10.7717/peerj.13875 (PMC9390326; doi:10.7717/peerj.13875)
Supplement: Supplemental Information 3 [file peerj-10-13875-s003.pdf]

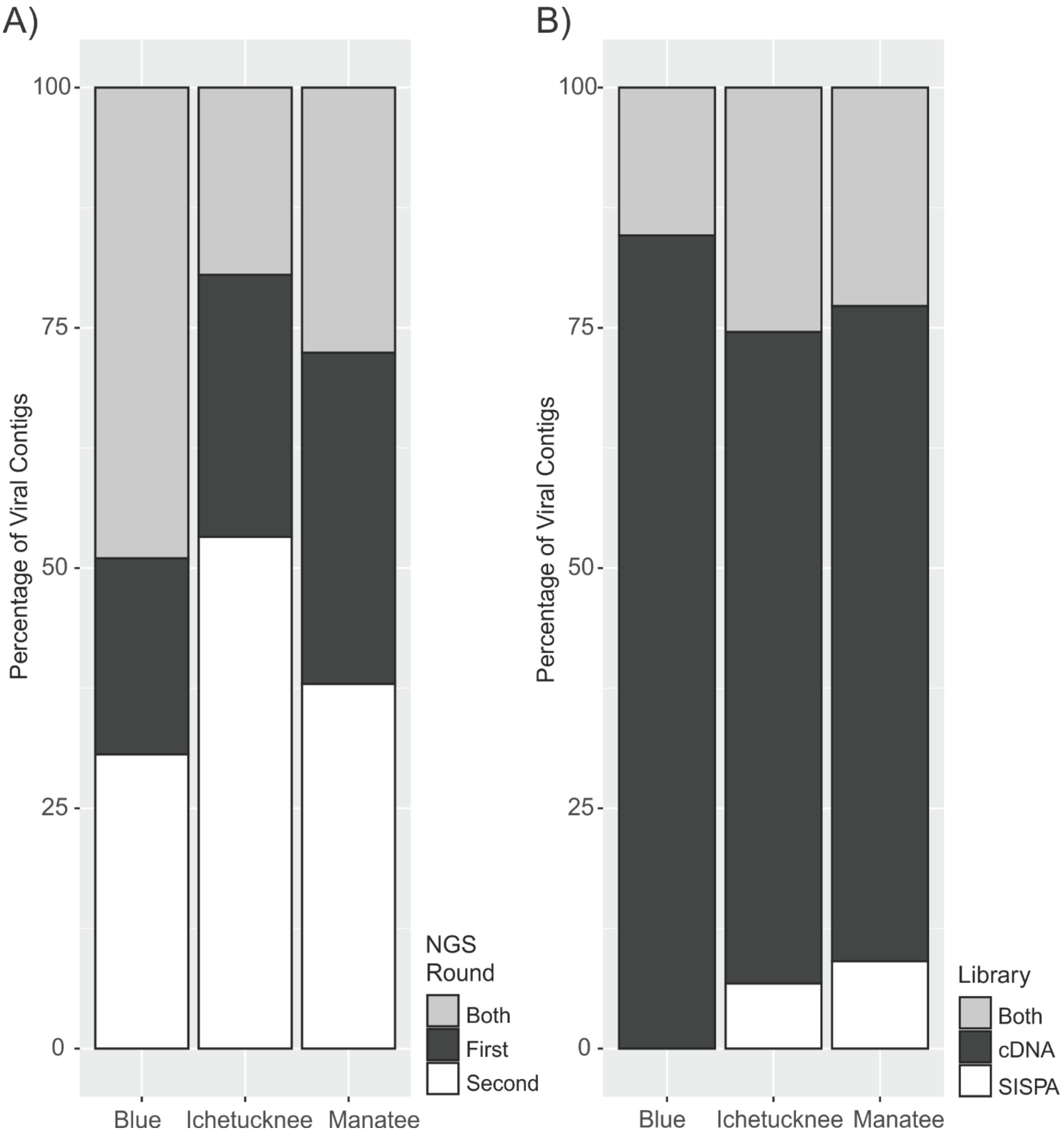

Figure S1. Distribution of viral contigs detected from pooled macrophyte samples through two rounds of next generation sequencing (NGS). Panel A shows the distribution of viral contigs based on the NGS round (Table 2). Panel B shows the distribution of contigs for Round 2 of NGS, which included samples prepared similarly (Table 2). Two approaches were used to prepare NGS libraries in Round 2. One approach targeted viral cDNA without preamplification for NGS (cDNA libraries), while the second approach exploited sequence-independent single-primer amplification (SISPA), which includes amplification prior to NGS library preparation.

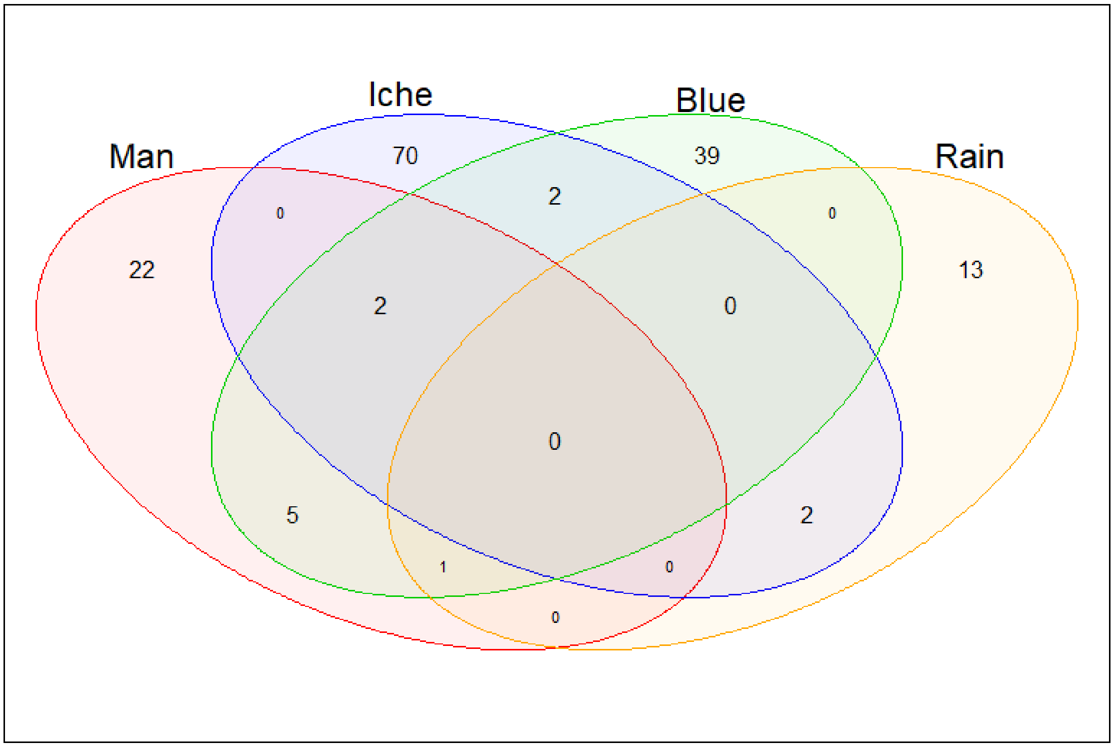

Figure S2. Venn diagram showing overlap among viral contigs detected in pooled macrophyte samples from four freshwater springs, including Manatee (red; n = 30), Ichetucknee (blue; n = 76), Blue (green; n = 49), and Rainbow (yellow; n = 16). Venn diagram was constructed using the *limma* R package (Ritchie et al. 2015).

Reference:

Ritchie ME, Phipson B, Wu D, Hu Y, Law CW, Shi W, and Smyth GK. 2015. *limma* powers differential expression analyses for RNA-sequencing and microarray studies. *Nucleic Acids Research* 43: e47. https://doi.org/10.1093/nar/gkv007.

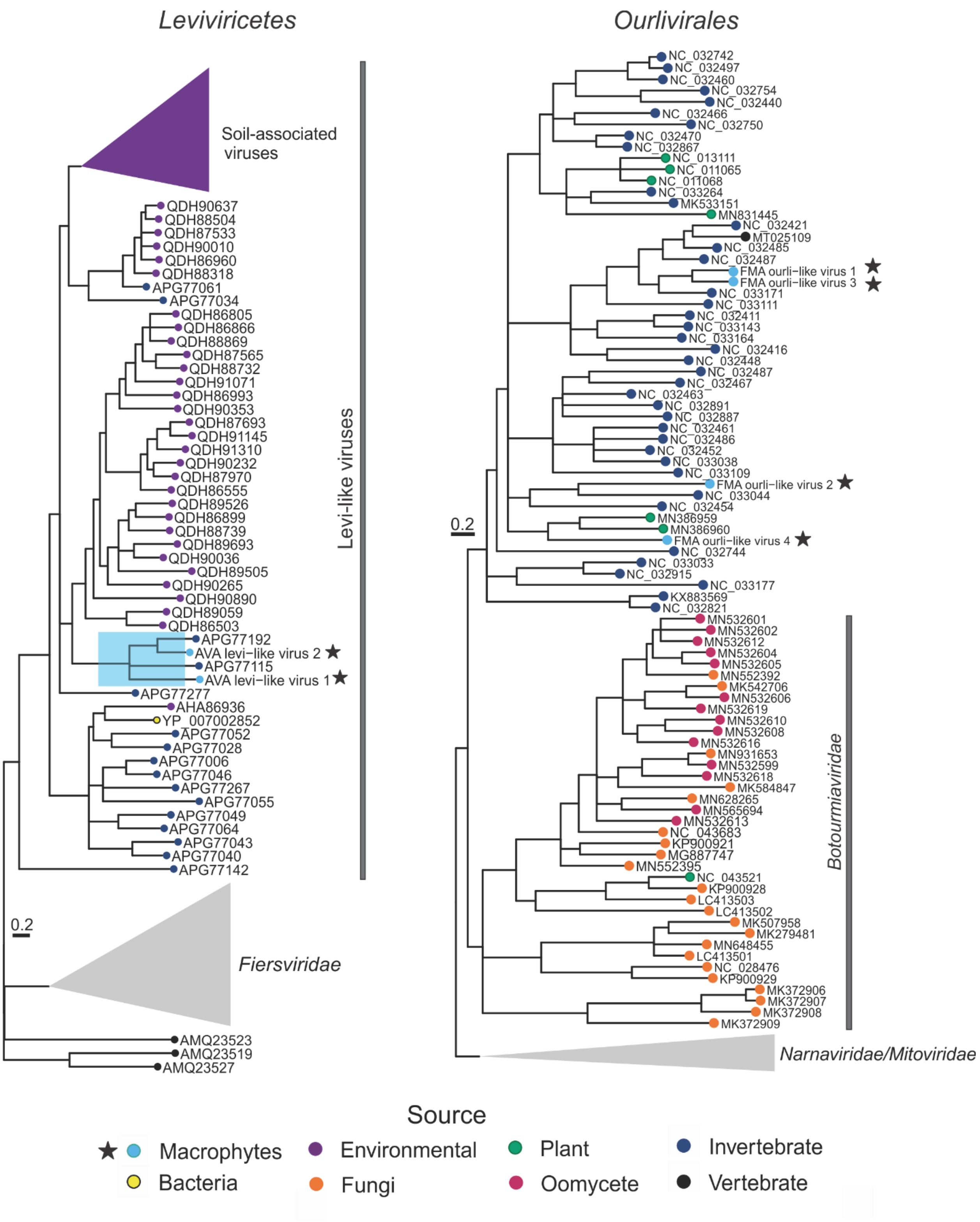

Figure S3. Midpoint-rooted maximum likelihood phylogenetic trees showing relationships among members of the *Leviviricetes* class (left) and *Ourlivirales* order (right) based on predicted RdRp amino acid sequences. Freshwater macrophyte associated (FMA) sequences are highlighted with a star. Clade highlighted in blue points to levi-like viruses from aquatic organisms. Branches with <70% aLRT support values were collapsed.

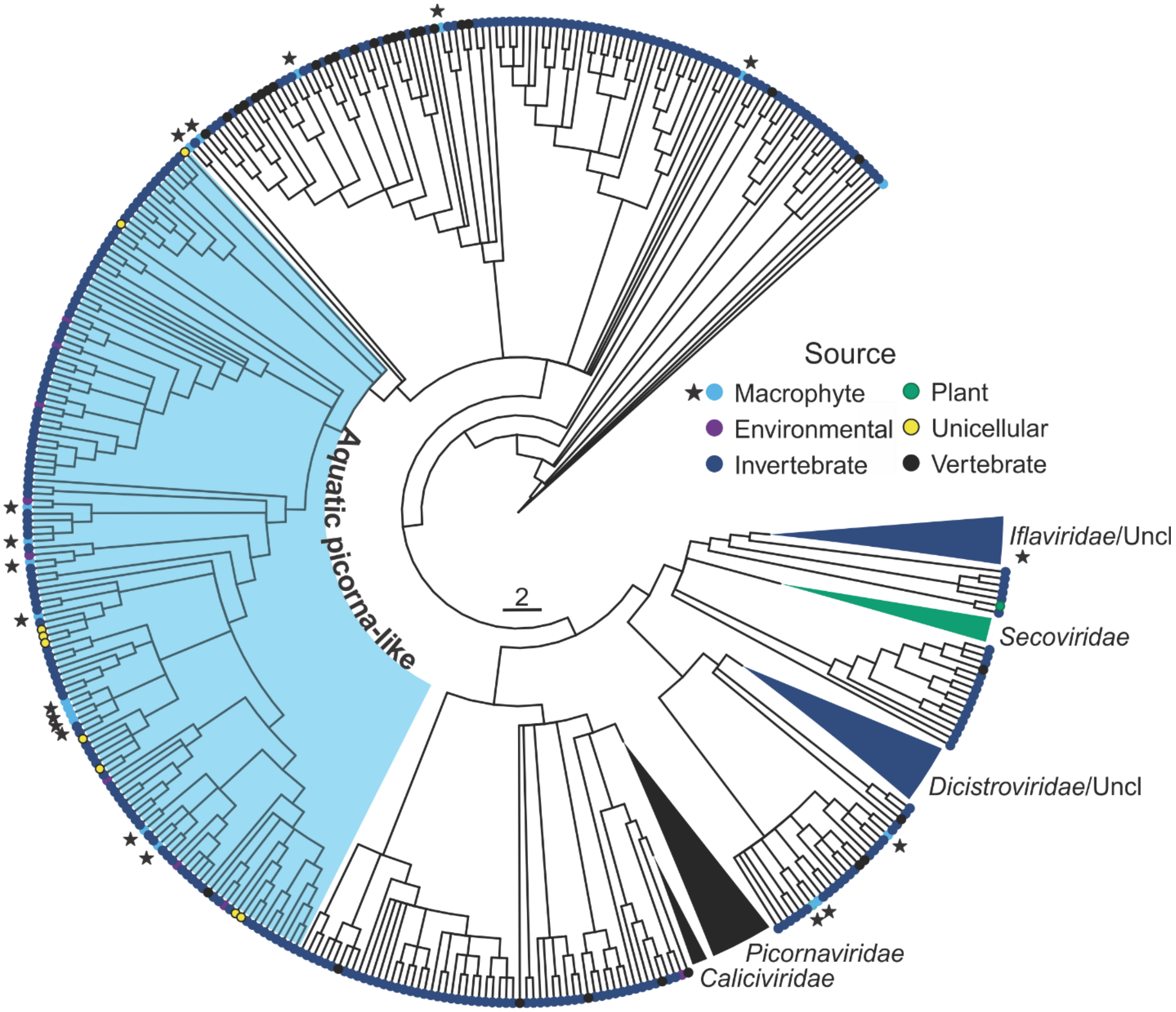

Figure S4. Unrooted maximum likelihood phylogenetic tree for members of the order *Picornavirales* based on predicted RdRp amino acid sequences. Freshwater macrophyte associated (FMA) sequences are highlighted with a star. The unicellular source group refers to single-celled, eukaryotic photosynthetic organisms (e.g., diatoms). Clade highlighted in blue points to the ‘aquatic picorna-like viruses’. Branches with <70% aLRT support values were collapsed.

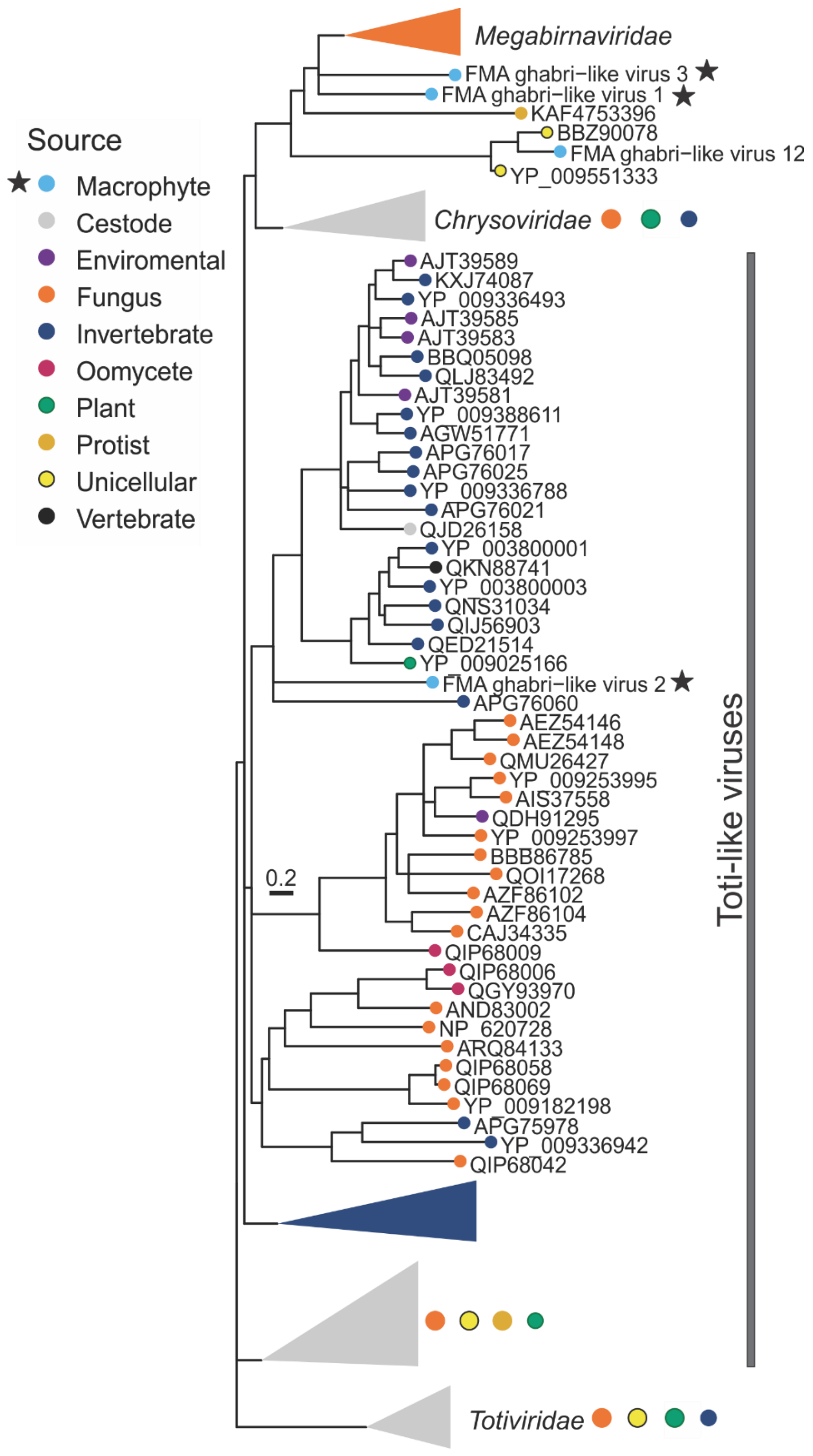

Figure S5. Midpoint-rooted maximum likelihood phylogenetic tree of members of the order *Ghabrivirales* based on predicted RdRp amino acid sequences. The freshwater macrophyte associated (FMA) sequences are highlighted with a star. The unicellular source group refers to single-celled, eukaryotic photosynthetic organisms (e.g., diatoms). Branches with <70% aLRT support values were collapsed.

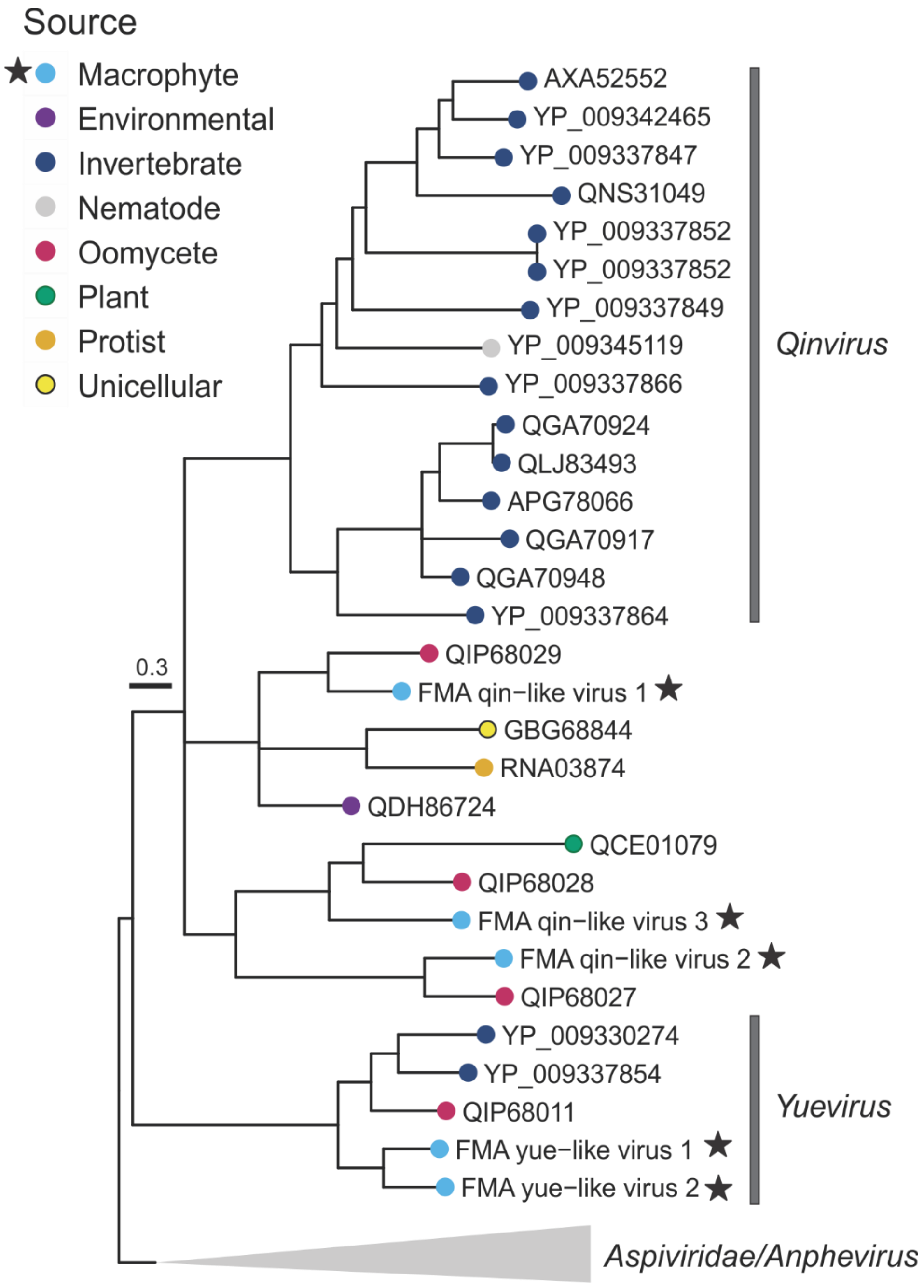

Figure S6. Midpoint-rooted maximum likelihood phylogenetic tree of members of the orders *Muvirales* (family *Qinviridae*) and *Goujianvirales* (family *Yueviridae*) based on predicted RdRp amino acid sequences. The freshwater macrophyte associated (FMA) sequences are highlighted with a star. The unicellular source group refers to single-celled, eukaryotic photosynthetic organisms (e.g., diatoms). Branches with <70% aLRT support values were collapsed.

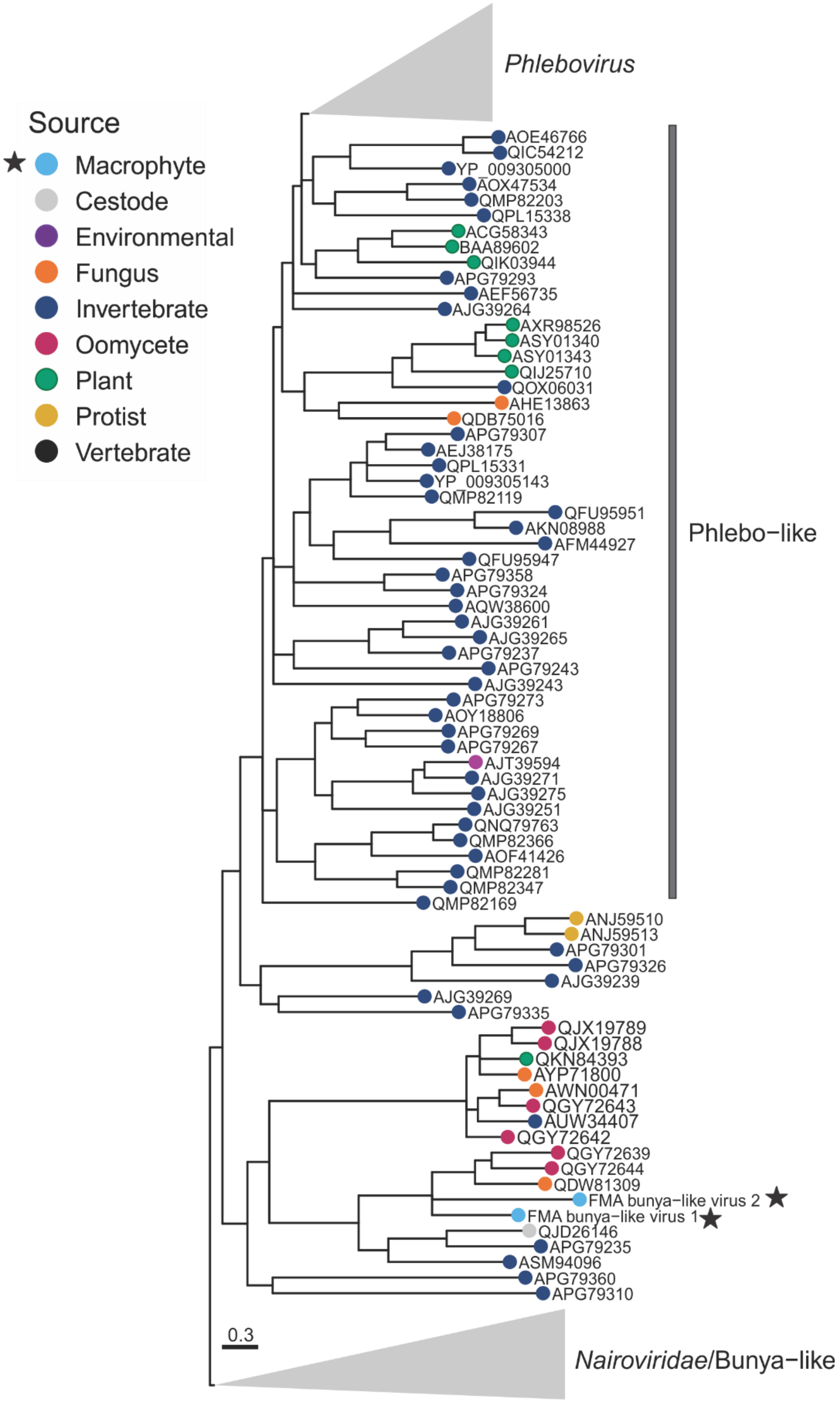

Figure S7. Midpoint-rooted maximum likelihood phylogenetic tree of members of the order *Bunyavirales* based on predicted RdRp amino acid sequences. The freshwater macrophyte associated (FMA) sequences are highlighted with a star. Branches with <70% aLRT support values were collapsed.

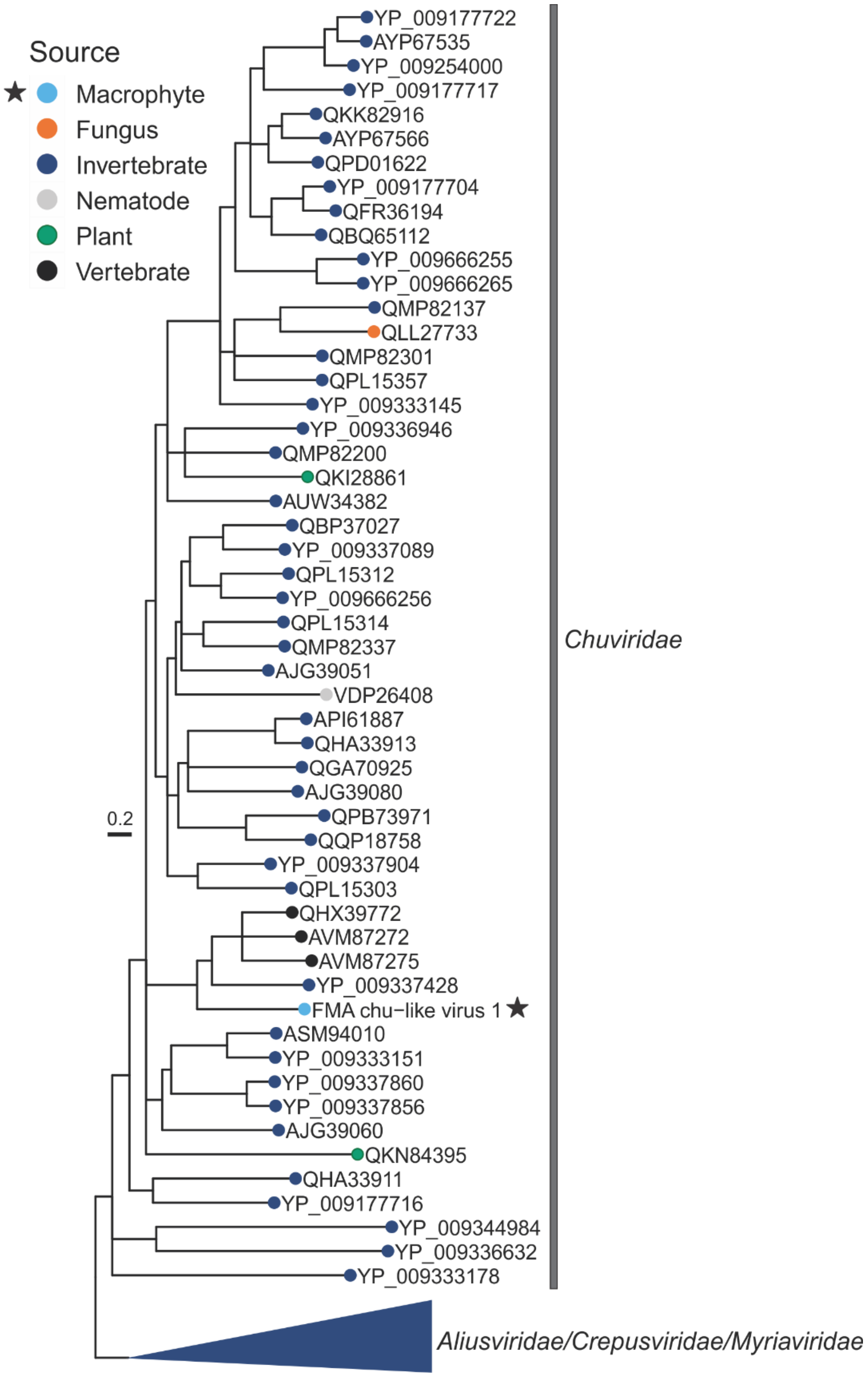

Figure S8. Midpoint-rooted maximum likelihood phylogenetic tree of members of the order *Jingchuvirales* based on predicted RdRp amino acid sequences. The freshwater macrophyte associated (FMA) sequence is highlighted with a star. Branches with <70% aLRT support values were collapsed.
